# Investigation of Association between Susceptibility to Leprosy and SNPs inside and near the *BCHE* Gene of Butyrylcholinesterase

**DOI:** 10.1155/2012/184819

**Published:** 2012-02-22

**Authors:** Henrique J. P. Gomes, Ricardo L. R. Souza, Flávia Costa Prevedello, Marcelo Távora Mira, Eleidi A. Chautard-Freire-Maia

**Affiliations:** ^1^Department of Genetics, Federal University of Paraná, P.O. Box 19071, 81531-980 Curitiba, PR, Brazil; ^2^Core for Advanced Molecular Investigation, Medical School, Pontifical Catholic University of Paraná, Imaculada Conceição, 1155, 80215-901 Curitiba, PR, Brazil

## Abstract

Leprosy is a chronic disease caused by *Mycobacterium leprae* and affects the skin and the peripheral nervous system. Butyrylcholinesterase is coded by the *BCHE* gene, and the atypical allele (*70G*; rs1799807) has been investigated as a leprosy risk factor, with conflicting results. The present study estimated the frequencies of variants of rs1799807 and of five additional SNPs at the *BCHE* gene or near it: rs1126680, rs1803274, rs2863381, rs4440084, and rs4387996. A total of 167 patients and 150 healthy controls were genotyped by TaqMan PCR. Significantly higher allelic (*70G*) and genotypic (*70DG*) frequencies in rs1799807 were found in the patient group, with odds ratio (OR) of 6.33 (1.40 to 28.53) for the heterozygote. This finding was replicated in a comparison of the cases against a control group of 361 blood donors. The present data suggest that the atypical BChE variant may predispose to leprosy *per se*.

## 1. Introduction

Leprosy is a granulomatous, chronic infectious disease that, in spite of its ancient origin, still affects thousands of people around the globe. It is caused by the Gram-negative bacteria *Mycobacterium leprae* and injures predominantly the skin and the peripheral nervous system, leading to sensory loss in the skin, muscle weakness, and, often, hand and foot permanent disabilities. The WHO classifies leprosy in two clinically distinct groups: paucibacillary leprosy, characterized by five or fewer skin lesions and negative bacilloscopy, and multibacillary leprosy, which includes patients with six or more skin lesions and positive bacilloscopy. Individuals with the paucibacillary form usually present a strong cell-mediated response (Th1 type), activating NK cells and macrophages that provide a more efficient response against the bacteria. Individuals with multibacillary leprosy show humoral response (Th2 type), which suppresses macrophages, increasing the chances of systemic infection [1, 2].

The genetic background of the host influences the type of damage caused by the immune responses against an etiologic agent. It is well known that leprosy is a complex disease with still unknown environmental and genetic risk factors. Many candidate genes concerned with susceptibility to infection per se, clinical manifestation, and reversal reaction have been studied to date, such as non-MHC genes* VDR *(12q13.11) [3]; *NRAMP1* (2q35) [4, 5], *TLR2* (4q32) [6], *PARK2,* and *PACRG* (6q25-q26) [7], and MHC genes *DQB1*, *DQA1*, *DRB1 *[8], *MICA *[9], *TNFA *[10], and *C4B *[11]. In the present study, the candidate is the *BCHE *gene that codes for butyrylcholinesterase.

Human butyrylcholinesterase (BChE; EC 3.1.1.8; OMIM 177400) is a serum enzyme produced by the liver that hydrolyses esters of choline and other esters. BChE has been associated with lipid metabolism and xenobiotics detoxification [12, 13]. More than 70 variants of the *BCHE* gene, located at chromosomal region 3q26.1-q26.2, have already been described [14]. The atypical variant *70G* (209 *A* > *G*, rs1799807) codes for a BChE resistant to the hydrolysis of the muscle relaxant succinylcholine and may cause prolonged apnea after its administration [15]. Thomas et al. [16], based on the observation that some patients with leprosy presented prolonged apnea after the administration of succinylcholine, searched for a relationship between leprosy and BChE and showed association of the atypical BChE variant (*70G*) with this disease. However, data on this association are controversial: significantly higher frequencies of *70G *in patients when compared to controls were found again [17], whereas other studies failed to replicate this result [18, 19], possibly due to the lack of accuracy of the enzyme inhibition method of phenotyping. Here, we present results of an association study between leprosy and *BCHE* variants, including *70G*, characterized by DNA genotyping.

## 2. Materials and Methods

### 2.1. Samples

 A case-control approach was applied, including 167 nonconsanguineous patients and two independent control samples: the first was composed of 150 healthy individuals (contact controls) reporting previous history of exposure to leprosy through current or past cohabitation with leprosy affected individuals and no consanguinity with patients; the second control group was composed of blood donors recruited from the same geographic area and ethnic background as the cases. Patients were classified according to the WHO criteria [1]: 116 with multibacillary and 51 with paucibacillary leprosy. Patients and contact controls are from the Brazilian state of Paraná, being predominantly from Caucasian origin (76.3 ± 3.5%; 68.0 ± 3.8%, resp.), representing the ethnic distribution of this state. The control group of male Euro-Brazilian blood donors (age range between 18 to 30 years) was described elsewhere [20] and is the only Brazilian population sample with *BCHE* SNPs data determined by DNA analysis.

### 2.2. Laboratory Methods

 DNA was extracted from whole blood by salting out [21]. Tag SNPs were selected from the HapMap Project website (http://www.hapmap.org), each representing a different bin of linkage disequilibrium and having a minimum allele frequency (MAF) of 5% in the Caucasian samples. Following this criterion, two SNPs upstream (rs2863381 and rs4440084), three intragenic (rs1126680, *G–116A*; rs1799807, *D70G*; rs1803274, *A539T*), and one downstream the *BCHE* gene (rs4387996) were selected for genotyping.

 Genotyping was performed by fluorescence-based allelic discrimination as implemented in the ABI 7500 TaqMan platform. A 10 *μ*L solution containing 1.5 *μ*L of DNA solution (20 ng/*μ*L), 0.3 *μ*L of the TaqMan genotyping kit, 5.0 *μ*L of TaqMan universal PCR Master Mix, and 3.2 *μ*L of ultrapure water was submitted to PCR (45 cycles at 50°C for 2 min, 95°C for 10 min, 95°C for 15 secs of denaturation, and 60°C for 1 min of hybridization and extension).

 SNP rs1803274 (*A539T*) was also genotyped by PCR-SSCA for quality control. This SNP was amplified by PCR (35 cycles at 94°C, 48°C, and 72°C for 1 min each; final extension at 72°C for 10 min): 1 *μ*L of DNA (about 100 ng), 9 *μ*L of PCR super MIX (Invitrogen), and 10 pmol of each primer (P45 and P43 [22]). A volume of 10 *μ*L of PCR product was added to 10 *μ*L of the denaturation solution (95% formamide, 0.25% bromophenol blue, 0.25% xylene cyanole, 10 mM EDTA, and 10 mM NaOH), submitted to 94°C (5 min), and kept chilled on ice until application in a polyacrylamide gel (8%), followed by silver nitrate staining [23].

### 2.3. Statistical Analysis

 Frequency distributions, means ± standard errors, *t*-tests, and *χ* ^2^ tests were calculated using Statistica for Windows (Statsoft, Inc., 2000; http://www.statsoft.com). Arlequin 3.0 [24] was used to test for the Hardy-Weinberg equilibrium (HWE) and for estimating haplotype frequencies by maximum likelihood, using an expectation-maximization (EM) algorithm for genotypic data with unknown gametic phase. Linkage disequilibrium (LD) was estimated by the *r* ^2^ parameter as implemented in Haploview 4.2 [25]. Odds ratios were also calculated [26]. SPSS 13.0 for Windows (IBM; SPSS Inc., 1989–2004; http://www.spss.com) was used for forward (Wald) stepwise multiple logistic regression analyses. Bonferroni's correction for multiple testing was used to adjust *P* values, when necessary.

## 3. Results and Discussion

**Figure 1:**
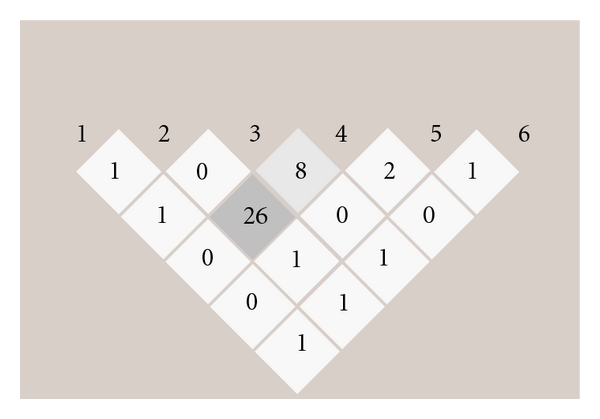
Values of *r* ^2^ (%) obtained from Haploview for pairs of the studied SNPs in the total sample of patients plus contact controls: rs4387996 downstream the *BCHE* gene (1); rs1126680 (2), rs1799807 (3), and rs1803274 (4) in the *BCHE* gene; rs4440084 (5) and rs2863381 (6) upstream the *BCHE* gene.

All markers were in HWE in patient and contact control samples.  Figure 1 shows pairwise *r* ^2^ estimates of LD for all six studied SNPs in the samples of patients and contact controls; highest *r* ^2^ value was 26%, confirming independence of all tag SNPs genotyped in this study.  Table 1 shows frequency distributions of allele and genotype data for all tag SNPs. Significant statistical differences were found between patients and contact controls for genotypic (*χ* ^2^ = 6.05; *P* = 0.014) and allelic (*χ* ^2^ = 5.90; *P* = 0.015) frequency distributions of SNP rs1799807: 7.88 ± 2.10% of heterozygotes (*70DG*) and 3.94 ± 1.01% of the *70G *allele in patients; 1.33 ± 0.94% of heterozygotes and 0.67 ± 0.57% of the *70G *allele in contact controls. The odds ratio (OR) was 6.33 (CI_(95%)_ from 1.40 to 28.53) for the *70DG* heterozygote. Pauci and multibacillary patients did not differ on *70G *or *70DG *frequencies, suggesting an impact of this variant over the early events of interaction between host and pathogen that result in infection *per se*, that is, the disease independent of its clinical form. Based on previous reports [16, 17] of association of the atypical variant with higher risk of susceptibility to leprosy, our initial hypothesis was that the frequency of this variant would be higher in patients than in controls. In view of this, Bonferroni's correction was not used for this case. Statistically significant difference between patients and contact controls was also found for allele frequencies of the SNP rs4387996 (Table 1) located downstream the *BCHE* gene (*χ* ^2^ = 5.07; *P* = 0.024). However, this significance did not resist Bonferroni's correction (*P* > 0.10).

 Patients and contact controls differ in sex composition (*χ* ^2^ = 5.20; *P* < 0.05) and mean age (*t* = 5.10; *P* = 5.72 ×10^−5^): the M/F ratio and mean age of the patients were 1.3 and 55.24 ± 1.37 years and of the controls were 0.8 and 46.06 ± 1.18 years, respectively. Since both gender and age are well-known risk factors for leprosy, these covariables were included, as independent variables, in a forward stepwise multiple logistic regression analysis in which leprosy was the dependent variable (Table 2). The strategy revealed three significant independent variables: median age, SNP rs4387996, and SNP rs1799807. In the presence of the age effect, association analysis revealed that the *A* allele of rs4387996 is protective, whereas the risk of the *70DG* genotype for acquiring leprosy is 5.18 times higher than that of the *70DD *genotype, confirming our previous analyses. The rs4387996 association was significant in both additive (Table 2, *P* = 0.021) and dominant models for allele *A* (*GG* versus *GA*+*AA; P* = 0.041; data not shown).

To confirm the findings for the *70DG* genotype, data from the case group was compared with genetic information obtained previously for a sample of 361 Euro-Brazilian blood donors of Curitiba [20], which presented 3.60% of heterozygotes and 1.80% of *70G* frequency. Again, statistically significant differences for genotype (*χ* ^2^ = 4.45; *P* < 0.05) and allele (*χ* ^2^ = 4.36; *P* < 0.05) frequencies were found. Estimate of OR for this second comparison was 2.29 (CI_(95%)_ from 1.04 to 5.05). The blood donor sample is appropriated for comparison, given the similar Euro-Brazilian origin of the cases, and the result clearly reinforces our hypothesis. Of note, the OR found in the comparison of cases against blood donor controls is lower than the one found for cases against contact controls. This may be due to the selection criterion for the contact control that may have led to a sample with a higher frequency of individuals presenting innate resistance to leprosy, as they have more likely been exposed than the blood donor controls and yet did not develop the disease. Furthermore, the contact controls report no consanguinity with any leprosy patient. Unfortunately, DNA data of the blood donor control group were not available for marker rs4387996, also associated with leprosy in the primary comparison.

To further advance on the understanding of the impact of *BCHE* variants over leprosy susceptibility, we performed a haplotypic analysis, involving all haplotypes with frequency > 5% in at least one of the samples (Table 3). Significantly higher frequency of the [*T*; *C*; *G*; *A*; *G*; *A*] haplotype was found in contact controls when compared to cases (*P* = 0.023 after Bonferroni's correction, OR = 0.34; CI_(95%)_ from 0.16 to 0.72). Although genotypes were of unknown phase and haplotype frequencies had to be estimated [24], the frequencies of this haplotype are expected to be rather accurate, considering that the EM algorithm usually presents very reliable performances for haplotype estimation [27]. Furthermore, both the total number of gametes (contact controls: 268; patients: 297) and the estimated haplotype numbers for contact controls (*N* = 25) and patients (*N* = 10) reinforce the accuracy of this frequency inference.

This protective haplotype differs from the most frequent one observed among cases ([*T*; *G*; *G*; *A*; *G*; *G*], 25.07%) only at positions corresponding to SNPs rs4440084 and rs4387996. These two variants (*C; A*) are found in the cis conformation in 10.92% and in 18.86% of haplotypes in patients and contact controls, respectively (*χ* ^2^ = 8.93; *P* = 0.003). The OR value for this comparison is 0.49 (CI_(95%)_ 0.30–0.79) and may indicate a protective effect that is two times higher than that of the haplotypes-bearing variants *G* and *G* in these respective SNPs. Considering that the rs4440084 SNP was not significant in the regression analysis (Table 2), it is possible that the protective effect of this haplotype is only due to the *A* variant of SNP rs4387996. However, the SNP independent variables in the regression analyses were the genotypes, as haplotypes were not known individually.

If the present data referring to the atypical variant (*70G*) is not due to linkage disequilibrium with other causal genetic variants, it is possible that the atypical variant of BChE may predispose to leprosy, although the physiological mechanism remains unclear. One possible hypothesis would be a relation of *70G* with the acetylcholine- (ACh-) mediated immune response. Activated leucocytes release small quantities of ACh [28], which acts like autocrine and paracrine signaling factors, mediating the contact with target cells. Moreover, when ACh is produced, it acts as part of an anti-inflammatory parasympathetic reaction, inhibiting the nuclear action of NF-*κ*B and stopping the production of proinflammatory citokynes as TNF, IL-1, IL-6, IL-8, and HMGB1, leading to the interruption of the inflammatory process [29]. Therefore, high levels of ACh are likely to cause a decrease in the inflammatory response. It is possible to speculate that this decrease in the inflammatory process can lead to a less competent host response to *M. leprae*. Acetylcholinesterase (AChE) activity is inhibited by excess of ACh [30]. Once AChE is inhibited, BChE becomes responsible for ACh hydrolysis [31]. The usual (*70D*) BChE variant is able to accomplish this function, but the atypical variant (*70G*), which has less affinity to the ACh substrate, could interfere in this regulation, failing in reducing ACh levels, consequently preventing the inflammatory response and contributing to the acquisition of leprosy.

 Considering that SNP rs4387996 independently associated with leprosy in our population sample is intergenic, it is difficult to infer a functional role. However, one possible hypothesis is that the variants (*G*, *A*) are in linkage disequilibrium with variants of the *SLITRK3* gene located downstream the *BCHE *gene but in the same LD bin. The Slitrk3 protein encoded by this gene is a neuronal transmembrane protein that controls axons and dendrites outgrowth [32]. The association among *BCHE* variants and the *SLITRK3* gene is unclear, but it is possible that they may be markers of the influence of this gene in the neuronal development which, somehow, can protect the nerves from *M. leprae*.

The present study reveals two independent signals of association between leprosy and variants of the *BCHE* gene, including the known functional variant *70G* (rs1799807), establishing *BCHE *as a strong candidate gene for the control of leprosy susceptibility. It is not clear whether the association of the *70G* variant with leprosy is really functional or is due to linkage disequilibrium with a variant of another, unknown gene. However, it is valid to suppose that the functional impact of this SNP that can lead to a less efficient BChE may predispose to the disease by failing to degrade a supposed substrate, such as acetylcholine, whenever it should act upon it. This fact could disturb the immune response when mediated by acetylcholine, reducing the inflammatory process and increasing the susceptibility to leprosy.

## Figures and Tables

**Table 1:** Frequency distributions of allele and genotype data for the six studied SNPs in patients (*M*: multibacillary; *P*: paucibacillary) and contact controls, as indicated by the nucleotide base.

SNP^a^	Sample (*n*)	Genotypes (%)			Alleles (%)	
rs2863381		*TT*	*TC *	*CC *	*T *	*C*
	Patients (161)	58.39 ± 3.88	34.16 ± 3.74	7.45 ± 2.07	75.47 ± 2.41	24.73 ± 2.41
	M (111)	62.16 ± 4.60	30.63 ± 4.38	7.21 ± 2.45	77.48 ± 2.82	22.52 ± 2.82
	P (50)	50.00 ± 7.07	42.00 ± 6.98	8.00 ± 3.84	71.00 ± 4.53	29.00 ± 4.53
	Controls (143)	44.06 ± 4.15	49.65 ± 4.18	6.29 ± 2.03	68.88 ± 2.73	31.12 ± 2.73
rs4440084		*GG*	*GC*	*CC*	*G *	*C*
	Patients (157)	36.31 ± 3.84	49.68 ± 3.99	14.01 ± 2.77	61.15 ± 2.75	38.85 ± 2.75
	M (107)	37.38 ± 4.67	44.86 ± 4.81	17.76 ± 3.69	59.81 ± 3.35	40.19 ± 3.35
	P (50)	34.00 ± 6.70	60.00 ± 6.93	6.00 ± 3.36	64.00 ± 4.80	36.00 ± 4.80
	Controls (146)	33.56 ± 3.91	52.74 ± 4.13	13.70 ± 2.85	59.93 ± 2.87	40.07 ± 2.87
rs1126680		*GG*	*GA*	*AA *	*G*	*A*
	Patients (163)	86.50 ± 2.68	13.50 ± 2.68	0.00 ± 0.00	93.25 ± 1.41	6.75 ± 1.41
	M (113)	87.61 ± 3.10	12.39 ± 3.10	0.00 ± 0.00	93.81 ± 1.58	6.19 ± 1.58
	P (50)	84.00 ± 5.18	16.00 ± 5.18	0.00 ± 0.00	92.00 ± 2.71	8.00 ± 2.71
	Controls (142)	86.49 ± 2.87	12.16 ± 2.74	1.35 ± 0.97	92.57 ± 1.58	7.43 ± 1.58
rs1799807^b^ (*D70G*)		*AA*	*AG *	*GG*	*A *	*G*
	Patients (165)	92.12 ± 2.10	7.88 ± 2.10	0.00 ± 0.00	96.06 ± 1.01	3.94 ± 1.01
	M (114)	92.11 ± 2.52	7.89 ± 2.52	0.00 ± 0.00	96.05 ± 1.30	3.95 ± 1.30
	P (51)	92.16 ± 3.76	7.84 ± 3.76	0.00 ± 0.00	96.08 ± 1.94	3.92 ± 1.94
	Controls (150)	98.67 ± 0.94	1.33 ± 0.94	0.00 ± 0.00	99.33 ± 0.57	0.67 ± 0.57
rs1803274 (*A539T*)		*GG*	*GA*	*AA*	*G *	*A*
	Patients (163)	67.48 ± 3.67	29.45 ± 3.57	3.07 ± 1.35	82.21 ± 2.13	17.79 ± 2.13
	M (113)	66.37 ± 4.44	31.86 ± 4.38	1.77 ± 1.24	82.30 ± 2.56	17.70 ± 2.56
	P (50)	70.00 ± 6.48	24.00 ± 6.04	6.00 ± 3.36	82.00 ± 3.84	18.00 ± 3.84
	Controls (149)	68.45 ± 3.81	28.86 ± 3.71	2.68 ± 1.32	82.89 ± 2.18	17.11 ± 2.18
rs4387996		*GG*	*GA*	* AA*	*G *	*A*
	Patients (162)	47.53 ± 3.92	45.06 ± 3.90	7.41 ± 2.06	70.06 ± 2.55	29.94 ± 2.55
	M (112)	47.32 ± 4.71	44.64 ± 4.70	8.04 ± 2.57	69.64 ± 3.06	30.36 ± 306
	P (50)	48.00 ± 7.07	46.00 ± 7.05	6.00 ± 3.36	71.00 ± 4.54	29.00 ± 4.54
	Controls (148)	37.16 ± 3.97	48.65 ± 4.11	14.19 ± 2.86	61.49 ± 2.83	38.51 ± 2.83

^a^Most frequent alleles in Caucasians from the HapMap: rs2863381 (*T*), rs4440084 (*G*), rs1126680 (*G*), rs1799807 (*A*), rs1803274 (*G*), and rs4387996 (*G*).

^b^Significant statistical differences between patients and controls (in bold) for genotype frequencies (*P* = 0.014; *χ* ^2^ = 6.05 after the Yates correction) and for allele distributions (*P* = 0.015; *χ* ^2^ = 5.90 after Yates correction).

**Table 2:** Results from a forward stepwise multiple logistic regression in which leprosy (0 = contact control, 1 = patient) was the dependent variable.

Independent variables^a^	B ± S.E.	Wald	df	*P*	Odds ratio (95% C.I.)
Median age	0.91 ± 0.25	13.03	1	0.000	2.48 (1.51 to 4.05)
SNP rs4387996	−0.45 ± 0.20	5.34	1	0.021	0.64 (0.44 to 0.93)
SNP rs1799807	1.64 ± 0.79	4.34	1	0.037	5.18 (1.10 to 24.33)
Constant	−2.19 ± 0.97	5.07	1	0.024	0.11

^a^Median age (≤51 = 1, >51 = 2); SNP rs4387996 (*GG* = 1, *GA* = 2, *AA* = 3); SNP rs1799807 (70*GG* = 1, 70*DG* = 2). Other independent variables were not significant: rs2863381; rs4440084; rs1126680; rs1803274 and sex.

**Table 3:** Haplotype^a^ frequencies compared between contact control (C) and patient (P) samples.

		SNPs^b^				Samples (%)		*χ* ^2^; *P* (Bonferroni's correction)
rs2863381	rs4440084	rs1126680	rs1799807 (*D*70G)	rs1803274 (*A*539*T*)	rs4387996	C	P	
*T*	*G*	*G*	*A*	*G*	*G*	24.9	25.1	n.s.
*T*	*G*	*G*	*A*	*G*	*A*	12.0	13.8	n.s.
*T*	*C*	*G*	*A*	*G*	*G*	11.6	18.5	5.28; 0.0216 (0.151)
*T*	*C*	*G*	*A*	*G*	*A*	9.5	3.4	8.62; 0.0033 (0.023)
*C*	*C*	*G*	*A*	*G*	*G*	8.9	3.8	6.69; 0.0097 (0.068)
*C*	*C*	*G*	*A*	*G*	*A*	8.1	6.5	n.s.
*C*	*G*	*G*	*A*	*G*	*G*	7.1	6.5	n.s.

**^a^**Selected on the basis of frequency higher than 5% at least in one of the samples. ^b^Most frequent nucleotides in Caucasians from the HapMap: rs2863381 (*T*), rs4440084 (*G*), rs1126680 (*G*), rs1799807 (*A*), rs1803274 (*G*), and rs4387996 (*G*).
